# Modifications of the endosomal compartment in peripheral blood mononuclear cells and fibroblasts from Alzheimer's disease patients

**DOI:** 10.1038/tp.2015.87

**Published:** 2015-07-07

**Authors:** F Corlier, I Rivals, J Lagarde, L Hamelin, H Corne, L Dauphinot, K Ando, J-C Cossec, G Fontaine, G Dorothée, C Malaplate-Armand, J-L Olivier, B Dubois, M Bottlaender, C Duyckaerts, M Sarazin, M-C Potier, Clinical ImaBio3 team, Dr Amer Alnajjar-Carpentier, Dr Michel Logak, Dr Sara Leder, Dr Dominique Marchal, Dr Hélène Pitti-Ferandi, Dr Hélene Brugeilles, Dr Brigitte Roualdes, Dr Agnes Michon

**Affiliations:** 1 UPMC University Paris 06, UMRS 1127, Sorbonne Universités, Paris, France; 2 INSERM U 1127, Paris, France; 3 ICM Research Centre, CNRS UMR 7225, Paris, France; 4 Équipe de Statistique Appliquée, ESPCI ParisTech, PSL Research University, INSERM UMRS 1158, Paris, France; 5 Neurologie de la Mémoire et du Langage, Service de Neurologie, Université Paris Descartes, Sorbonne Paris Cité, INSERM UMR S894, Centre Hospitalier Sainte Anne, Paris, France; 6 INSERM UMRS 938, Laboratoire Système Immunitaire et Maladies Conformationnelles, Hôpital Saint-Antoine, Paris, France; 7 Université Pierre et Marie Curie, Université Paris 6, Centre de Recherche Saint-Antoine, Hôpital Saint-Antoine, Paris, France; 8 Laboratoire de Biochimie et Biologie Moléculaire, UF Oncologie—Endocrinologie—Neurobiologie, Hôpital Central, Centre Hospitalier Universitaire, Nancy, France; 9 UR AFPA—USC 340, Equipe BFLA, Université de Lorraine, Nancy, France; 10 Institut de la mémoire et de la maladie d'Alzheimer, IMMA, Hôpital de la Pitié-Salpêtrière, AP-HP, Paris, France; 11 CEA (MB), DSV, Institut d'Imagerie Biomédicale, Service Hospitalier Frédéric Joliot, Orsay, France; 12 Laboratoire de Neuropathologie Escourolle, Hôpital de la Pitié-Salpêtrière, AP-HP, Paris, France

## Abstract

Identification of blood-based biomarkers of Alzheimer's disease (AD) remains a challenge. Neuropathological studies have identified enlarged endosomes in post-mortem brains as the earliest cellular change associated to AD. Here the presence of enlarged endosomes was investigated in peripheral blood mononuclear cells from 48 biologically defined AD patients (25 with mild cognitive impairment and 23 with dementia (AD-D)), and 23 age-matched healthy controls using immunocytochemistry and confocal microscopy. The volume and number of endosomes were not significantly different between AD and controls. However, the percentage of cells containing enlarged endosomes was significantly higher in the AD-D group as compared with controls. Furthermore, endosomal volumes significantly correlated to [C^11^]PiB cortical index measured by positron emission tomography in the AD group, independently of the *APOE* genotype, but not to the levels of amyloid-beta, tau and phosphorylated tau measured in the cerebrospinal fluid. Importantly, we confirmed the presence of enlarged endosomes in fibroblasts from six unrelated AD-D patients as compared with five cognitively normal controls. This study is the first, to our knowledge, to report morphological alterations of the endosomal compartment in peripheral cells from AD patients correlated to amyloid load that will now be evaluated as a possible biomarker.

## Introduction

An estimated 35 million people worldwide suffer from dementia, and this number, predicted to double every 20 years, will rise to more than 65 million in 2030 and more than 115 million in 2050.^1^ Alzheimer's disease (AD) is the most common cause of dementia. New proposals for the diagnosis of AD combine clinical tools and biological markers such as cerebrospinal fluid (CSF) biomarkers and amyloid positron emission tomography (PET) imaging for identifying the underlying AD pathophysiological process. This approach allows establishing a clinical diagnosis of AD at the stage of mild cognitive impairment (MCI), before dementia, but needs invasive and expansive tools.^2^

Autopsy still remains the only fully reliable diagnosis. AD is neuropathologically characterized by extracellular amyloid plaques composed of β-amyloid peptides (Aβ) and intracellular neurofibrillary tangles constituted of hyperphosphorylated tau protein.^3^ Although the degree of tauopathy correlates strongly with cognitive decline in AD, the aggregation of Aβ has a critical role as early trigger, and recent longitudinal PET imaging studies indicated that cerebral Aβ deposition precedes the clinical symptoms of AD by a decade or more.^4, 5^

One of the main challenges associated with the diagnosis of AD is the identification and validation of blood-based biomarkers. Among different tracks, neuropathological and clinical data suggested the presence of enlarged endosomes as a candidate cellular biomarker in the blood.

Indeed dysregulation of the endolysosomal compartment has been found in the brain of AD patients.^6, 7^ Neurons bearing enlarged endosomes were identified as the earliest morphological change observed in post-mortem brains from patients with sporadic AD before the onset of clinical symptoms.^8, 9^ Although familial AD cases with mutations in the amyloid precursor protein gene harboured the same endosomal phenotype, cases with mutations in the presenilin genes did not.^10^ Enlarged endosomes were also observed in individuals with Down syndrome (DS) who are at high risk for AD (45% of DS people have AD at 60 years against ~3% in the general population).^11^ In DS, enlarged endosomes have been identified not only in neuronal cells but also in peripheral cells such as peripheral blood mononuclear cells (PBMCs), lymphoblastoids and fibroblasts.^12, 13^

AD genome-wide association studies suggest a crucial role of several proteins involved in membrane trafficking such as PICALM, SORL1, BIN1.^14^ In addition, several endolysosomal proteins such as EEA1, Rab3, Rab7, LAMP1 and LAMP2 were found increased in the CSF of AD patients.^15^ These data suggest that endosomal dysmorphologies could be associated with amyloid pathology in AD and could thus be detected in blood cells from AD patients at an early stage of the disease.

In this study, we applied the immunocytochemical method that we developed earlier for analysing the endosomal compartment of PBMCs from DS patients^13^ to isolated PBMCs from 48 biologically confirmed AD patients (25 with mild cognitive impairment (AD-MCI) and 23 at the dementia stage (AD-D)) and 23 cognitively normal controls, with no evidence of preclinical AD (negative for the amyloid imaging). Results were confirmed in fibroblasts from six independent AD-D patients and five cognitively normal controls. We hypothesised that the endosomes in cells from AD would be different from controls, even at the MCI stage.

## Materials and methods

### Participants

PBMCs were analysed from 71 subjects, enrolled in the ImaBio3 study (*NCT*: NCT01775696). Forty-eight AD patients with biologically confirmed AD were included according to the following criteria: (1) predominant and progressive episodic memory impairment, characterized by low free recall not normalized with cueing (free recall >19/48 and total recall <41/48 in the Free and Cued Selective Reminding Test, FCSRT), associated or not with other cognitive impairments; (2) biological evidence of AD pathophysiological process as defined by CSF AD biomarker profile and/or significant amyloid retention on PET with [C11]- labelled Pittsburgh Compound B ([C11]PiB). A CSF AD biomarker profile was defined as score <0.8 for the ratio of Aβ 42/tau, calculated with the formula Aβ 42/[240+(1.18 × total tau)].^16^ Significant fixation of [C11]PiB on PET was defined by a global cortical index higher than 1.4.^17^ We excluded subjects who had clinical or neuroimaging evidence of focal lesions, severe cortical or subcortical vascular lesions on magnetic resonance imaging and severe depression.

Patients were stratified according to disease severity assessed by the Clinical Dementia Rating scale (CDR): 25 patients had a score of 0.5 representing AD-MCI with isolated episodic memory impairments and only moderated impact on the activities of daily living, and 23 patients had a CDR scale score >0.5 representing AD-D.

Twenty-three healthy elderly control subjects were selected according to the following criteria: (1) MMSE (Mini Mental State Examination) ⩾27 and normal neuropsychological testing; (2) no history of psychiatric or neurologic conditions, (3) no evidence of focal lesion or severe cortical or subcortical vascular lesions on magnetic resonance imaging.

To improve diagnostic accuracy, all the subjects had at least one 12-month follow-up to validate the AD diagnosis according to their clinical evolution, or the absence of cognitive decline for controls.

The study was conducted by the Assistance Publique des Hôpitaux de Paris (PHRC-0053-N) and was approved by the Ethics Committee of Pitié-Salpêtrière Hospital. All the subjects provided written, informed consent before participating.

### Analysis of fibroblasts

Fibroblasts were obtained independently from a regional PHRC (Projet Hospitalier de Recherche Clinique) in 2003 coordinated by the Centre Hospitalo-Universitaire of Nancy (France). Eleven additional participants without a known family history of AD were enrolled: six patients with sporadic AD-D (mean age: 78.5±7.77, MMSE <25) and five healthy volunteers (mean age: 70.8±8.01, MMSE=30). Skin biopsies taken from the upper arm were explanted and grown in DMEM-glutamax supplemented with 10% fetal bovine serum, 100 units ml^−1^ penicillin and 100 μg ml^−1^ streptomycin (Gibco BRL, Life Technologies, Saint Aubin, France, European Division) at 37 °C in humidified air with 5% CO_2_. Experiments on AD and control fibroblasts were performed at the same stage of doubling in culture (<12 passages).

Participants were selected in accordance with local ethic recommendations: CCPPRB (Comité Consultatif de Protection des Personnes dans la Recherche Biomédicale) de Lorraine n° 04-03-01.

For both groups, all the subjects provided written informed consent before participating.

### Biological markers

#### CSF biomarker analysis

For the Imabio3 study, CSF samples obtained by lumbar puncture were processed with the same procedures described previously^18^ to obtain CSF levels of total tau, phosphorylated tau at threonine 181 (P-Tau) and amyloid-β peptide 1–42 (amyloid-β_42_) by using enzyme-linked immunosorbent assay kits (Innogenetics), according to the manufacturer's instructions. All the operators were masked to clinical information. CSF biomarkers were available for 36/48 AD patients, among whom 20/25 were AD-MCI. Controls did not have lumbar punction for ethical reasons.

#### [C^11^]PiB PET imaging procedures

For the Imabio3 study, PET imaging with [C^11^]PiB was performed in 19 control subjects and in 43/48 AD patients, among whom 23/25 were AD-MCI. The method was the same as previously described.^17^ In summary, a global cortical index was defined by the mean standard uptake value ratio (with the cerebellum as the reference region) of the following cortical regions: (1) frontal cortex, by grouping the orbitofrontal, polar prefrontal and dorsolateral cortex; (2) anterior cingulate; (3) medial cingulate; (4) posterior cingulate; (5) precuneus; (6) occipital cortex, by grouping the calcarine cortex, occipital cortex and cuneus; (7) temporal cortex, by grouping the anterior and lateral temporal cortex; (8) hippocampus; and (9) parietal cortex, by grouping the inferior and superior parietal cortex and the parietotemporal junction.

#### Measures of enlarged endosomes

**PBMC isolation from blood samples:** For each participant, 10 ml blood was collected in heparin-coated tubes (Vacutainer, Beckton-Dickinson, Franklin Lakes, NJ, USA). Blood samples were diluted in one volume of phosphate-buffered saline and PBMCs isolated by centrifugation on a 1077 g ml^−1^ ficoll gradient (15 ml prefilled filtered Leucosep PANCOLL tubes, PAN Biotech, Aidenbach, Germany) following the manufacturer's instructions. After centrifugation, PBMCs were re-suspended in 200 μl of reduced serum culture medium (Gibco OPTI-MEM) and incubated at 37 °C, 5% CO_2_ for 1 h, then rinsed and fixed for 12 min in 4% cold paraformaldehyde and rinsed in 10 ml phosphate-buffered saline.

**Culture of fibroblast:** Fibroblasts were grown directly on microscope coverslips in 24-well plates, in DMEM supplemented with 10% FCS and 100 μg ml^−1^ penicillin+streptomycin, for 24 to 48 h. Cells were fixed before reaching confluence in 4% cold paraformaldehyde for 15 min and rinsed three times with phosphate-buffered saline. The number of passages was between 3 and 12 at the time of analysis.

**Immunocytochemistry:** Paraformaldehyde-fixed PBMCs were incubated with 2% normal goat serum for 20 min and permeabilized 20 min in Triton 0.1% X-100. For staining of EEA1, cells were re-suspended in 1:100 solution of Polyclonal antibody (Cell signalling, Rabbit-anti-Human #2411) in 2% normal goat serum at 5 °C overnight. Cells were rinsed in phosphate-buffered saline twice and incubated with 1:500 Alexa-488®-conjugated Goat-anti-Rabbit antibody (A-11034, Invitrogen) in 2% normal goat serum, for 2 h at room temperature. Slides were mounted by mixing 10 μl of re-suspended cells in 10 μl mounting medium (SouthernBiotech, fluoromount-G) and sealed after one night air drying at room temperature.

For fibroblasts, EEA1 immuno-staining was performed following the same protocol directly on coverslips with an additional staining of nuclei before the last wash using DAPI for 2.5 min. Mounting was performed by applying coverslips upside down on a drop of mounting medium and sealed after one night air drying at room temperature.

**Confocal imaging:** Immunofluorescent labelling was observed under an upright confocal microscope (Olympus Fluoview Fv1000) using a × 63 (NA 1.40) apochromatic objective. All PBMC images were performed at the same magnification. (Voxel size for PBMC: XY=0.073 μm, *Z*=0.25 μm). Due to the large size of fibroblasts, we applied a lower magnification (Voxel size: XY=0.094 μm, *Z*=0.25 μm). Approximately 20 cells per subject for PBMCs and 15 cells per subject for fibroblasts were chosen randomly on the slides and scanned individually by defining the upper and lower position of the scanning device and by fixing the space between slides to 250 nm (the actual depth of field of the objective) to sample the whole cell volume for subsequent three-dimensional image treatment.

**Image analysis of the endosomes:** The three-dimensional reconstructed images were analysed applying a wavelet-based detection method^19^ implemented as a plugin spot detector in Icy software^20^ (http://icy.bioimageanalysis.org.) as described earlier for the analysis of cells from DS individuals.^13^ The size was expressed as the number of voxels in the detection, and when needed, converted in cubic micrometres (μm^3^) according to voxel size. Staining and image analysis were performed blindly by the same operator to guarantee anonymous and unbiased interventions at all levels of sample processing and to avoid inter-operator variability.

**Automated data processing:** Data extraction and analysis was automatized using MATLAB version 8.3.0.73043 (R2014a). The function selects relevant data in the single result files provided by Icy software for each cell, and implements a concatenated database of all subjects by classifying them automatically with respect to clinical information, relating every single detected endosome to the cell, as well as to the individual and his clinical information.

### Statistical analysis

The statistical analysis was performed with MATLAB's Statistics Toolbox Version 9.0 (R2014a).

#### Comparison of the endosome number and mean volume per cell

The endosome numbers and mean volumes per cell of the three groups, that is, the control group (23 patients, 459 cells), the MCI-AD group (25 patients, 497 cells) and the AD group (23 patients, 455 cells) were compared with an analysis of variance with a fixed group effect and a random individual effect. To normalize the distributions and to homogenize their variances, the analysis of variance was performed on the logarithm of the mean numbers and volumes.

#### Comparison of the mean endosome volume distribution between groups

To compare the distributions of the endosome volumes per cell in the three groups, we classified the mean volumes in size categories. The distributions being heavily skewed to the right (overall skewness and kurtosis of 2.7 and 16.6, respectively), we chose the median and ninth decile of the control group volumes as the limits of three size categories small, medium and large. The distributions of the mean endosome volumes per cell in the three size categories of the three groups were then compared with a *χ*^2^ test. Further *χ*^2^ tests were used to perform the three two-by-two comparisons between groups, that is, controls/AD, controls/MCI-AD and MCI-AD/AD; the *P*-values of the latter were adjusted for multiple testing using the Bonferroni correction.

As the analysis of variance had shown a significant individual effect on the mean endosome volume per cell, we used bootstrap sampling at the individual level (1000 samples were randomly drawn) to assess the stability of the distributions across the size categories and of the significance of the difference between the distributions in the control and AD groups.

#### Correlation of the mean endosome volume with the clinical data

Pearson's correlation was computed between the mean endosome volume of the patients (MCI-AD and AD) and their amyloid load measured by PiB retention. A generalized linear model was used to adjust for age and *APOE* genotype.

## Results

### Population demographics

Morphometric analyses of early endosomes (number and volume) in PBMCs were obtained from 48 patients with AD (23 AD and 25 MCI-AD) and 23 healthy controls at inclusion (Table 1).

There were no significant differences in sex and age across the AD-MCI, AD-D and control groups. As expected, there were significant differences across the diagnostic groups (MCI-AD and AD) for *APOE* ɛ4 status, amyloid burden and cognitive deficits (MMSE and CDR) with AD and MCI-AD groups having more *APOE* ɛ4 carriers and poorer performance on cognitive assessments as compared with healthy controls. Individual data are presented as Supplementary Material (Supplementary Table).

### Analysis of the mean endosome volume and number in PBMCs

The PBMCs from all patients with AD-D, AD-MCI and healthy controls were purified from fresh blood, fixed and their early endosomes stained by immunocytochemistry using an antibody against early endosome antigen 1 (EEA1). Z-stack images of ~20 cells for each individual were obtained under the confocal microscope.

**Figure 1:**
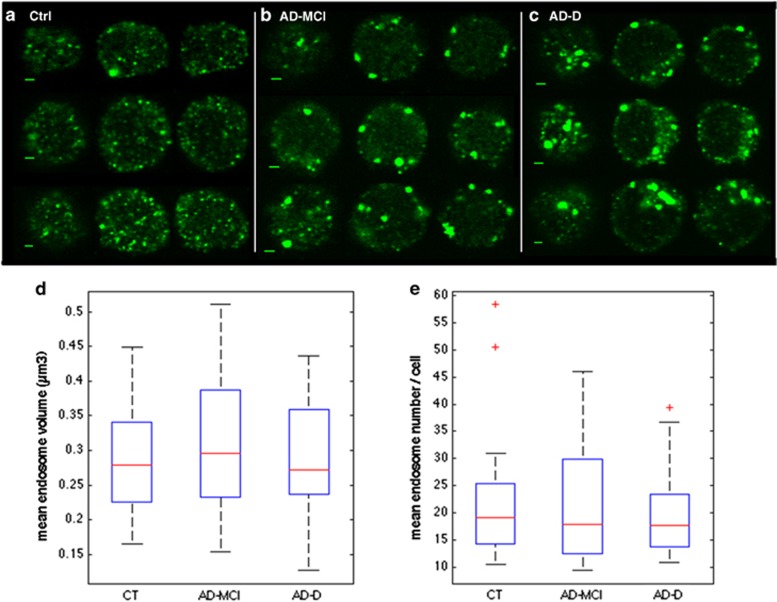
Endosomal abnormalities are present in peripheral mononuclear blood cells (PBMCs) from individuals with AD-MCI and AD-D. (**a**–**c**) Immunofluorescence confocal images showing EEA1-labelled early endosomes in representative PBMCs from cognitively healthy subjects (**a**), AD-MCI (**b**) and AD-D cases (**c**). Scale bar, 2 μm. Mean endosome volumes (MEVs) (**d**) and mean endosome numbers (MENs) (**e**) per PBMC are not significantly different between AD-D (455 cells), AD-MCI (497 cells) and controls (CT, 459 cells). *P*_group_=0.63 for MEN and 0.61 for MEV according to analysis of variance. AD-D, Alzheimer's disease with dementia; AD-MCI, Alzheimer's disease with mild congnitive impairment.

Figures 1a–c show representative images obtained from PBMCs of control, AD-MCI and AD-D individuals. Videos of three-dimensional reconstructed images are available as Supplementary Material (Supplementary Videos 1–3).

Endosomes present in the whole cell volume were detected with the spot detector plugin of Icy software.^20^ Volumes of individual endosomes were measured as voxels. A total of 459, 497 and 455 cells were analysed in the healthy control, AD-MCI and AD-D groups, respectively. For each individual, mean endosome number (MEN) and mean endosome volume (MEV) per cell were calculated. Figures 1d and e show boxplots of individual values for the healthy controls, AD-MCI and AD-D cases. Individual data are presented as Supplementary Material (Supplementary Table).

According to analysis of variance, MEN and MEV were not significantly different either between AD-MCI, AD-D and control groups (*P*_group_=0.63 for MEN and 0.61 for MEV) or between AD group (AD-MCI and AD-D) and controls. The MENs per cell were 22.47 for the controls, 20.12 for the AD-MCI and 21.92 for the AD-D. The MEVs per cell were 214.90, 214.81 and 233.83 voxels corresponding to 0.286 and 0.311 μm^3^ in the control/AD-MCI and AD-D groups, respectively.

To verify between-day variability of endosome size and number measurements for a given subject, we analysed blood samples drawn from the same individual with no cognitive decline at 1-month interval. No significant difference was found in MEV and MEN (Student's *t*-test, *P*=0.29 and 0.17, respectively, data not shown).

### Analysis of the endosome size distribution

**Figure 2:**
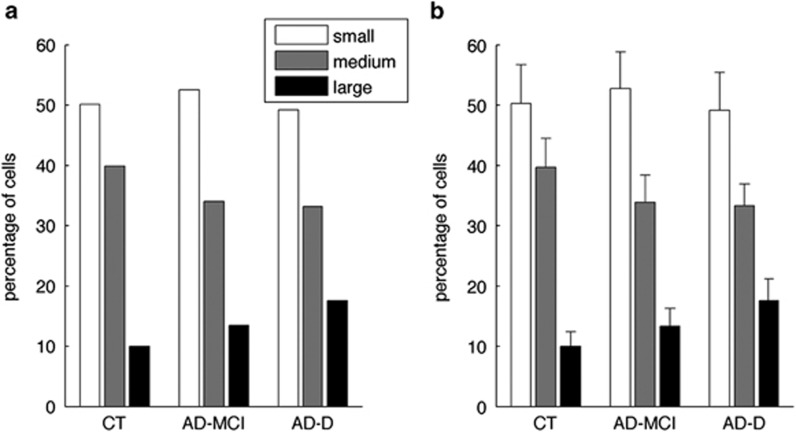
Percentage of PBMCs containing enlarged endosomes is significantly higher in AD-D as compared with control (CT) individuals. (**a**) Classification of cells in three subclasses according to their MEV (small/medium/large) and (**b**) mean percentages and standard deviations on 1000 bootstrap samples drawn at the individual level. AD-D, Alzheimer's disease with dementia; AD-MCI, Alzheimer's disease with mild congnitive impairment; MEV, mean endosome volume; PBMC, peripheral blood mononuclear cell.

We then categorized the 459, 497 and 455 cells from the healthy control, AD-MCI and AD-D groups, respectively, in three classes according to their mean endosomal volume. Small, medium and large were defined as classes containing endosomes with mean volume below the median (<0.256 μm^3^; small), between the ninth decile and the median (0.256 to 0.412 μm^3^; medium) and above the ninth decile (>0.412 μm^3^; large) in the control group. Figure 2a shows the percentage of cells with MEV of the three classes for the three groups. A higher percentage of cells with larger MEV were found in the AD groups (13.5 and 17.4% for the AD-MCI and the AD-D, respectively) as compared controls (10.0%). Endosome size distributions were significantly different for the AD-D and the control groups (Bonferroni corrected *P*=6.4 10^−3^ of *χ*^2^ test).

We confirmed the stability of these size distributions by drawing 1000 bootstrap samples at the individual level: the percentage of cells with large MEV was significantly higher in the AD groups as compared with control individuals (Bonferroni corrected *P*<0.05) in 98% of the bootstrap samples. Means and standard deviations on the bootstrap samples are shown in Figure 2b. These data suggest that the size distribution of endosome was distinct between healthy controls and AD groups.

Analysis of samples from the same control individual drawn at 1-month interval did not show any difference in the distribution of mean endosomal volume (*P*=0.12 of *χ*^2^ test, data not shown).

### Correlation between the mean endosomal volume per cell and the cortical amyloid burden assessed by [C^11^]PiB PET

We then wondered what could cause the increase in the percentage of cells with bigger endosomes in the AD-MCI and AD-D cases. Since Aβ levels and *APOE* ɛ4 status have been shown to be associated with the increase in the size of early endosomes observed in neurons from AD patients,^9^ we analysed the potential correlation between amyloid burden, the *APOE* status and the mean endosomal volumes in the AD groups.

**Figure 3:**
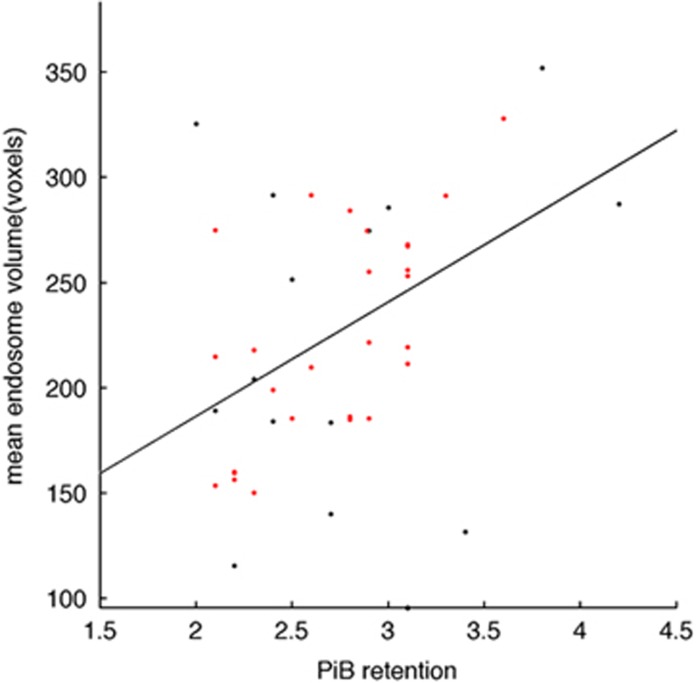
Correlation between mean endosomal volume in AD patients (AD-MCI and AD-D) and their PiB retention. Black dots correspond to non-*APOE* ɛ4 carriers and red dots to *APOE* ɛ4 carriers; *r*=0.682, *P*=0.0001 and *r*=0.415, *P*=0.008 for all AD patients (black and red dots) and *APOE* ɛ4 carriers only (red dots) after adjusting for age and *APOE* ɛ4. AD-D, Alzheimer's disease with dementia; AD-MCI, Alzheimer's disease with mild congnitive impairment; PiB, Pittsburgh Compound B.

Pearson's correlations were used to test the level of overlap between endosomal volume and global amyloid burden measured by [C^11^]PiB retention. We found a significant positive correlation between MEV and global amyloid burden in 43 AD-MCI and AD-D patients (*r*=0.415, *P*=0.008 after adjustment for age and *APOE* ɛ4; Figure 3). High correlation was found in the precuneus, a region with greatest levels of amyloid load^21^ (*r*=0.402, *P*=0.018 after adjustment for age and *APOE* ɛ4).

In addition, we found a positive correlation (0.88) between MEV and the *APOE* ɛ4 status (0, 1 or 2 alleles), although this correlation was below statistical significance (0.16, data not shown). MEV was also more strongly correlated to PiB retention (*r*=0.682, *P*=0.0001 as compared with *r*=0.415, *P*=0.008, see above) when we analysed AD-MCI and AD-D *APOE* ɛ4 carriers only (28 individuals, Figure 3, dots in red).

Finally, we did not find any correlation between the MEV and other parameters such as the levels of Aβ, tau or P-tau in the CSF, age and sex (data not shown).

### Analysis of the endosomal compartment in fibroblasts from AD-D individuals

**Figure 4:**
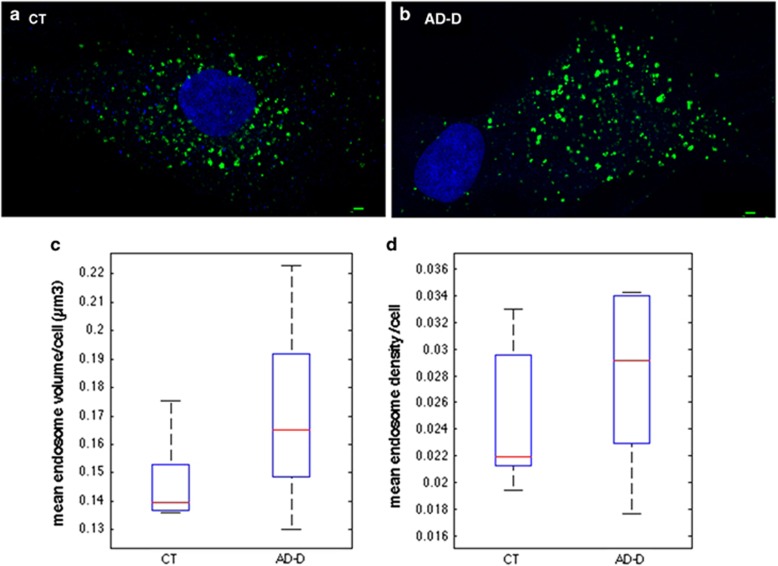
Endosomal abnormalities are present in fibroblasts from individuals with AD-D. Immunofluorescence confocal images showing EEA1-labelled early endosomes (green) in representative fibroblasts from cognitively healthy subjects (**a**) and AD-D cases (**b**). Nuclei are labelled in blue. Scale bar, 2 μm. (**c**) Mean endosome volumes per fibroblast are not significantly different between control (CT) individuals (0.147±0.016 μm^3^) and AD-D patients (0.170±0.034 μm^3^; Wilcoxon *P*=0.18). (**d**) Comparison of the mean endosomal densities per fibroblast (that is, portion of the cell volume occupied by endosomes) showed no significant difference between control individuals (0.0249±0.005) and AD-D patients (0.0279±0.006; Wilcoxon *P*=0.46). AD-D, Alzheimer's disease with dementia; EEA1, early endosome antigen 1.

To confirm the presence of abnormally larger endosomes in peripheral cells from AD patients, we analysed the endosomal compartment of fibroblasts isolated from six independent AD-D patients (90 cells) and five age-matched controls (76 cells). Figures 4a and b show representative images obtained from fibroblasts of control (a) and AD-D individuals (b). Instead of reporting the number of endosomes per cell, which would be biased by the particularly high variability of shape and size among fibroblasts, we compared the density of endosomes per cell, the number of endosomes per cell divided by its volume obtained by calculating the convex hull of all endosomes. As for PBMCs, neither the mean density nor the MEV was significantly different between AD-D and controls (Figures 4c and d).

**Figure 5:**
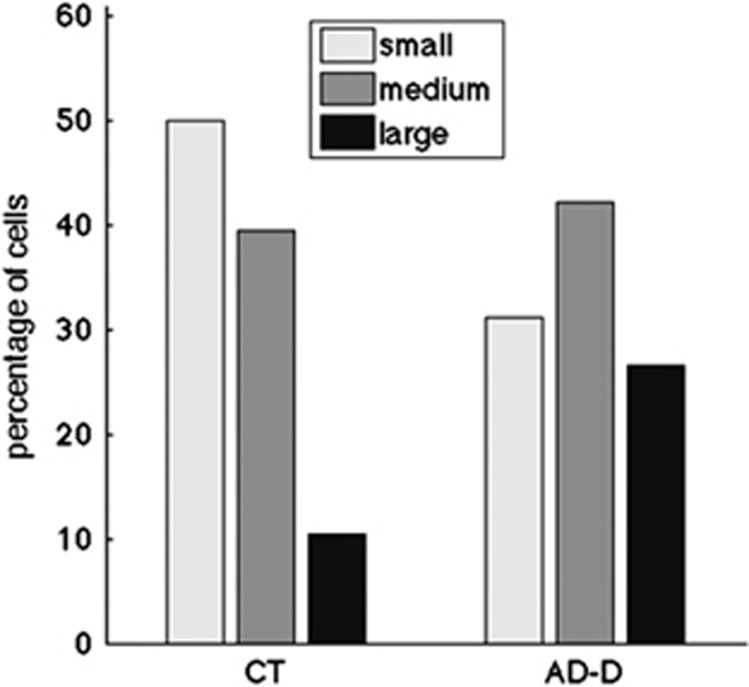
Percentage of fibroblasts containing enlarged endosomes is significantly higher in AD-D as compared with control (CT) individuals. Classification of cells in three subclasses according to their MEV (small/medium/large) showed significantly elevated percentage of cells with large MEV in AD-D patients and reduced percentage of cells with small MEV (*χ*^2^ test, *P*=0.0094). AD-D, Alzheimer's disease with dementia; MEV, mean endosome volume.

We then categorized the 76 and 90 cells from the healthy control and AD-D groups, respectively, in three classes according to their mean endosomal volume. Small, medium and large were defined as classes containing endosomes with mean volume below the median (<0.14 μm^3^; small), between the ninth decile and the median (0.141 to 0.19 μm^3^; medium) and above the ninth decile (>0.191 μm^3^; large) in the control group. Figure 5 shows the percentage of cells with MEV of the three classes: 50% small, 39.5% medium and 10.5% large for the controls and 31.1% small, 42.2%medium and 26.7% large for the AD-D. A significantly higher percentage of cells with larger MEV was found in the AD-D group as compared with controls (*χ*^2^ test, *P*=0.0094).

## Discussion

Previous studies which aimed at identifying blood biomarkers in AD were based on large longitudinal cohorts using top down approach.^22^ Here, we used a hypothesis-driven approach based on a very well-defined population of AD patients. We applied strict inclusion criteria based on both positive clinical/cognitive and pathophysiological markers (CSF biomarkers and [C^11^]PiB PET). More importantly, all healthy controls had negative cortical [C^11^]PiB retention, thus excluding presymptomatic AD. We focused our research on the analysis of the endosomal compartment, a putative cellular biomarker expected to be less sensitive to serum components.

Across the three very well-defined groups (AD-MCI, AD-D and controls), we found a significant increase in the percentage of cells containing enlarged endosomes in AD patients as compared with controls, even at an early stage of the disease (AD-MCI).

Importantly, we confirmed the presence of enlarged endosomes in fibroblasts from another small cohort of AD-D patients thus demonstrating the presence of endosomal abnormalities in a second peripheral cell type.

These results support the recent line of evidence showing an over-representation of genes involved in endocytosis as AD genetic risk factors.^23^ It will be interesting in a next study to include these genetic risk factors.

Enlarged endosomes have been previously identified in neuronal cells of AD-D, AD-MCI and DS patients as well as in peripheral cells from individuals with DS.^9, 12^ Changes of the endosomal compartment observed here in PBMCs were significantly correlated in the AD diagnostic groups with amyloid burden assessed by PET using [C^11^]PiB retention. Notably, this correlation was still valid after conditioning with age and *APOE* ɛ4 status. It thus appears that factors other than *APOE* ɛ4 allele and age likely contribute to the change observed in the morphology of early endosomes. Even though changes in the endosomal compartment of neuronal cells appear specific of AD and DS,^9^ it will be important to analyse the endosomal compartment of peripheral cells in other types of neurodegenerative diseases than AD or DS (present study and Cossec *et al.*^13^). At least no correlation was found between MEV of PBMCs from AD patients and their age suggesting that such changes in the endosomal compartment are not correlated to age.

Aβ has been linked to endosomal dysfunctions in AD and DS. Indeed enlarged endosomes in post-mortem brains of sporadic AD and DS patients contain Aβ.^9^ However, enlarged endosomes are present in DS brains before Aβ can be detected and conversely enlarged endosomes are absent in familial AD brains with mutations in presenilin1 overproducing Aβ.^9^ In DS fibroblasts, amyloid precursor protein C-terminal fragment (β-CTF) rather than Aβ appeared to be related to endosomal dysfunction.^24^ It will be interesting to compare the levels of β-CTF in fibroblasts from controls individuals and AD patients.

In PBMC from AD patients (MCI and D), we found that enlarged endosomes are more strongly correlated to amyloid burden when we stratified *APOE* ɛ4 carriers (Supplementary Figure 1). But since this correlation in AD-D and AD-MCI was still valid after conditioning with *APOE* genotype, the *APOE* genotype could participate nonexclusively in the formation of enlarged endosomes in peripheral cells.

The levels of plasma *APOE* may be important for the endososomal function as *APOE* ɛ4 carriers have lower levels of plasma APOE4 protein that could predispose them to AD.^25^ However, *APOE* genotype does not influence the metabolism of plasma Aβ peptides in young persons without memory deficits.^26^ In addition, *APOE* ɛ4- but not ɛ4+ AD participants showed positive relationships between plasma Aβ1–40/Aβ1–42 and PiB uptake.^27^

Macrophages are able to cross the blood–brain barrier and phagocytize cerebral Aβ while Aβ-engorged macrophages are adherent and cannot cross the blood–brain barrier back into the blood.^28^ We were unable to detect Aβ in PBMCs using immunocytochemistry (data not shown). Thus, the role of peripheral Aβ in enlarged endosomes found in PBMC remains unclear.

PBMCs are blood cells with a round nucleus including 70–90% lymphocytes (T cells, B cells and NK cells), 10–30% monocytes and few percentage of macrophages and dendritic cells. The higher percentage of PBMCs containing enlarged endosomes observed in AD and AD-MCI compared with healthy controls could be the result of either a change of the endosomal compartment in a subpopulation of cells or a change in a small percentage of all cell types. Additional experiments of co-labelling of large endosome bearing cells with selective markers, or cell sorting using a panel of antibodies including anti-EEA1 will be necessary to confirm one of these hypotheses. However, as fibroblasts also carry this enlarged-endosome phenotype, we believe that endosomal changes are more likely to be present in a small percentage of all cell types of PBMCs rather than in a specific subpopulation.

Inflammation could also be involved in endocytosis and the morphological changes of endosomes. As inflammation is a hallmark of AD and the neutrophil–lymphocyte ratio was shown to be elevated in AD, it will be interesting to test the correlation between endosomal volume and the neutrophil–lymphocyte ratio.^29^

We showed previously that higher cholesterol levels at the plasma membrane of cultured cells and neurons induced enlargement of early endosomes and subsequent increase of Aβ.^30, 31, 32^ It is also well admitted that cholesterol levels are increased in AD brains notably in membranes as well as in amyloid plaques.^33, 34, 35^ As neutral lipids have been found to be increased in a very high percentage of PBMCs in AD (85% compared with 7% in controls), and cholesterol-related genes are also differentially expressed in PBMC, it remains possible that cholesterol homeostasis could be involved in endosome changes observed in PBMCs in AD patients.^36, 37^

Nevertheless, it will be interesting to know whether some of the endolysosomal proteins that have been shown to be increased in the CSF of AD patients, including EEA1, are also changed in PBMC or plasma.^15^

In conclusion, in this study, we found morphological alterations of the endosomal compartment in blood cells (PBMCs) from sporadic biologically confirmed AD patients as well as in fibroblasts of clinically defined independent sporadic AD patients that may reflect amyloid pathology in the brain and could thus be a new peripheral and blood biomarker. Future studies will determine whether changes in the endosomal compartment in PBMCs and fibroblasts from the same patients are equally correlated to amyloid load, how they evolve during the time course of the disease and whether endosomal biomarkers, either cellular or plasmatic, can be identified.

## Figures and Tables

**Table 1:** Demographic and clinical data of analysed groups

	*Controls*	*AD-MCI*	*AD-D*
*PBMC analysis*			
Number of individuals (*N*)	23	25	23
Sex ratio (F/M)	13/10	14/11	12/11
Age	67±10.39 (51–86)	67.68±9.7 (52–82)	69.43 ±11.6 (51–91)
MMSE	29.9±0.92 (27–30)	23.7±3.19 (18–30)	15.43±4.9 (4–22)
CDR=0.5	—	25	—
CDR=1	—	—	23
*APOE* ɛ4/ -	2/23	10/25	7/23
ɛ4/ɛ4	0/23	7/25	5/23
[11]C-PIB PET scan	20/23	23/25	20/23
Lumbar puncture	—	20/25	16/23
Number of analysed cells	454	460	427
% Cells with small MEV	50.1	51.7	46.8
% Cells with medium MEV	39.9	34.8	35.8
% Cells with large MEV	10.0	13.5	17.4
			
*Fibroblast analysis*			
Number of individuals (*N*)	5	—	6
Sex ratio (F/M)	5/0	—	1/5
Age	70.8±8.01 (63–83)	—	78.5±7.77 (69–88)
MMSE	30		18±2.45 (16–21)
*APOE* ɛ4/ -	0/5	—	2/6
ɛ4/ɛ4	1/5	—	1/6
Number of analysed cells	76		90
% Cells with small MEV	50.0		31.1
% Cells with medium MEV	39.5		42.2
% Cells with large MEV	10.5		26.7

Abbreviations: AD-D, Alzheimer's disease with dementia; AD-MCI, Alzheimer's disease with mild congnitive impairment; CDR, Clinical Dementia Rating; MEV, mean endosome volume; MMSE, Mini Mental State Examination; PBMC, peripheral blood mononuclear cell; PET, positron emission tomography.
