# Supplementary material for: Modifications of the endosomal compartment in peripheral blood mononuclear cells and fibroblasts from Alzheimer's disease patients
Source: Transl Psychiatry. 2015 Jul 7;5(7):e595–. doi: 10.1038/tp.2015.87 (PMC5068716; doi:10.1038/tp.2015.87)
Supplement: Supplementary Information [file tp201587x1.doc]

**Supplemental Material**

Supplementary Video 1: Video of 3D reconstructed images of immunolabelled early endosomes (in green) in a PBMC from a Control subject.

Supplementary Video 2: Video of 3D reconstructed images of immunolabelled early endosomes (in green) in a PBMC from an AD-MCI subject.

Supplementary Video 3: Video of 3D reconstructed images of immunolabelled early endosomes (in green) in a PBMC from an AD-D subject.

Supplementary Table: Individual values of clinical parameters and endosomal volumes and numbers of the Controls, AD-MCI and AD-D subjects.
